# ENDOCRINE HEALTH: Nitrate May Feed Thyroid Disorders

**DOI:** 10.1289/ehp.118-2898875

**Published:** 2010-06

**Authors:** Bob Weinhold

**Affiliations:** **Bob Weinhold**, MA, has covered environmental health issues for numerous outlets since 1996. He is a member of the Society of Environmental Journalists

Thyroid cancer incidence has been rising substantially worldwide since at least the 1970s,^1^ but the reasons remain murky. Trend data are scant for two other thyroid diseases, hyperthyroidism and hypothyroidism, but incidence estimates suggest each is at least 5 times more common than thyroid cancer.^2^,^3^ Limited evidence has suggested substances such as polychlorinated biphenyls, polybrominated diphenyl ethers, bisphenol A, and triclosan may be playing a role in these disorders. Nitrate also may belong on that list of suspects, U.S. researchers now report.^4^

The new study is apparently the first to link nitrate ingestion and thyroid cancer. The authors found that the quartile of women who consumed drinking water containing an average concentration of nitrate–nitrogen^5^ (NO_3_–N) greater than 2.46 mg/L—about one-fourth the EPA maximum contaminant level of 10 mg/L—were 2.18 times more likely to have thyroid cancer than the bottom quartile, whose water contained less than 0.36 mg/L NO_3_–N. Consuming water containing more than 5 mg/L NO_3_–N for more than five years increased the risk slightly more. The women in the highest quartile of daily nitrate ingestion from food were 2.9 times more likely to have thyroid cancer than the quartile who consumed the least, and were 24% more likely to report having hypothyroidism. Nitrate in food dominated total nitrate intake.

The study was based on records from women who were followed from 1986 to 2004 as part of the larger Iowa Women’s Health study. The women were 55–69 years old at recruitment. The researchers had extensive self-reported demographic, behavioral, and medical data, but fewer data on overall diet and none on nitrate levels in individuals. Instead, they had to develop intake estimates based on local public water supply monitoring data and national food nitrate data. The small number of thyroid cancers—40 in the approximately 21,000 women analyzed—also is a limitation, although the findings were statistically significant.^6^

The numerous limitations make Lewis Braverman, a professor of medicine at the Boston University School of Medicine, somewhat skeptical of the conclusions. “The evidence is fairly soft,” he says. He notes, though, there has been some evidence globally of a nitrate-related association with hypothyroidism, goiter, and other thyroid disorders in subjects such as pregnant women and children exposed to far higher nitrate concentrations than those reported in the present study.^7^ Ongoing and planned studies, including one looking at men, may provide additional clues, says Mary Ward, lead author of the current study and a senior investigator at the National Cancer Institute.

Thyroid cancer is estimated to be the seventh leading site of new cancers in U.S. women, according to the American Cancer Society.^8^ Incidence is about three times higher in women than in men, similar to the pattern worldwide.^1^ Some experts are concluding that increased detection alone is not likely to be responsible for global increases.^9^

Synthetic fertilizer can be an important source of nitrate in water and food.^10^ The foods that often have the highest concentrations of nitrate, such as spinach, kale, and beets, often are touted for their nutritional benefits. The women in this study who were hardest hit by thyroid disorders tended to be better educated, physically active nonsmokers—a group Ward says is more likely to eat vegetables.
